# Efficient Production of 9,22-Dihydroxy-23,24-bisnorchol-4-ene-3-one from Phytosterols by Modifying Multiple Genes in *Mycobacterium fortuitum*

**DOI:** 10.3390/ijms25073579

**Published:** 2024-03-22

**Authors:** Suwan Han, Xiangcen Liu, Beiru He, Xinghui Zhai, Chenyang Yuan, Yixin Li, Weichao Lin, Haoyu Wang, Baoguo Zhang

**Affiliations:** 1 Laboratory of Biorefinery, Shanghai Advanced Research Institute, Chinese Academy of Sciences, No. 99 Haike Road, Pudong, Shanghai 201210, China; hansw@sari.ac.cn (S.H.); liucx@sari.ac.cn (X.L.); hebr2022@shanghaitech.edu.cn (B.H.); zhaixh@sari.ac.cn (X.Z.); yuanchy@shanghaitech.edu.cn (C.Y.); linwc@sari.ac.cn (W.L.); 2 University of Chinese Academy of Sciences, Beijing 100049, China; 3 School of Life Science and Technology, ShanghaiTech University, Shanghai 201210, China; wanghy2022@shanghaitech.edu.cn; 4 Department of Biology, Colby College, Waterville, ME 04901, USA; yli24@colby.edu

**Keywords:** C22 steroids, 9,22-dihydroxy-23,24-bisnorchol-4-enehp-3-one(9-OHBA), *Mycobacterium fortuitum*, phytosterol, *hsd4A*, *fadA5*

## Abstract

C19 steroids and C22 steroids are vital intermediates for the synthesis of steroid drugs. Compared with C19 steroids, C22 steroids are more suitable for synthesizing progesterone and adrenocortical hormones, albeit less developed. 9,22-dihydroxy-23,24-bisnorchol-4-ene-3-one(9-OHBA), due to its substituents at positions C-9 and C-22, is a beneficial and innovative steroid derivative for synthesizing corticosteroids. We focused on the C22 pathway in *Mycobacterium fortuitum* ATCC 35855, aiming to develop a productive strain that produces 9-OHBA. We used a mutant strain, MFΔ*kstD*, that knocked out *kstds* from *Mycobacterium fortuitum* ATCC 35855 named MFKD in this study as the original strain. Hsd4A and FadA5 are key enzymes in controlling the C19 metabolic pathway of steroids in *Mycobacterium fortuitum* ATCC 35855. After knocking out *hsd4A*, MFKDΔ*hsd4A* accumulated 81.47% 9-OHBA compared with 4.13% 9-OHBA in the strain MFKD. The double mutant MFKDΔ*hsd4A*Δ*fadA5* further improved the selectivity of 9-OHBA to 95.13%, and 9α-hydroxy-4-androstenedione (9-OHAD) decreased to 0.90% from 4.19%. In the end, we obtained 6.81 g/L 9-OHBA from 10 g/L phytosterols with a molar yield of 80.33%, which showed the best performance compared with formerly reported strains.

## 1. Introduction

Steroid-based drugs have a wide range of therapeutic uses, including regulating hormone levels and blood pressure and suppressing the inflammatory response [1,2,3]. C19 and C22 intermediates can be converted into steroid-based drugs using chemical methods [4,5,6]. Nowadays, the biotransformation of steroid-based drug intermediates from phytosterols attracts more attention due to its eco-friendliness and mild reaction conditions compared to the traditional chemical synthesis routine [7].

Phytosterols, consisting of β-sitosterol, campesterol, and stigmasterol, are accessible on the raw materials market and are often produced as a by-product or waste of the vegetable oil refining industry [8]. Thanks to their low cost and abundant availability, phytosterols are good substitutes for producing steroid-based drug intermediates. However, β-sitosterol, stigmasterol, and campesterol are only distinguished by one carbon atom or one double bond on the side chain, which brings about high costs for the separation of each component. Fortunately, Actinomycetes, especially mycobacteria, known for their outstanding abilities of incompletely degrading the different steroid components in phytosterols, have been industrially used to produce steroid-based drug intermediates and have also been genetically modified to accumulate steroid-based drug intermediates. Among these, technologies for the production of C19 intermediates consisting of 4-androstene-3,17-dione (AD), 1,4-androstadiene-3,17-dione (ADD), and 9α-hydroxy-4-androstenedione (9-OHAD) have been studied a lot [9,10,11,12,13,14]. By contrast, technologies for the production of C22 intermediates are less developed, which show advantages in synthesizing some progestin and adrenal hormones, such as progesterone, hydrocortisone, dexamethasone, and drospirenone [15,16,17]. 

The C22 steroid intermediates include 22-hydroxy-23,24-bisnorchol-4-en-3-one (BA), 22-hydroxy-23,24-bisnorchol-1,4-dien-3-one (1,4-BA), and 9,22-dihydroxy-23,24-bisnorchol-4-en-3-one (9-OHBA). Among these, 9-OHBA is a beneficial and innovative steroid derivative for synthesizing corticosteroids due to its hydroxyl group at positions C-9 and C-22. Using *M. neoaurum* ATCC 25795 (NwIB-XII) as a model, the deletion of *hsd4A* resulted in the production of 23,24-bisnorcholenic steroids [17]. Finally, *M. neoaurum* ATCC 25795 deleting *hsd4A*, *kstD1*, *kstD2,* and *kstD3* accumulated 10.25–10.72 g 9-OHBA from 40 g phytosterols with a molar yield of 30–32% [17]. Later in 2021, the double deletion of *hsd4A* and *fadA5* in a *M. neoaurum* DSM 44074 *kstd*-null strain was found to significantly accumulate 3.58 g/L 9-OHBA with the cultivation of 5 g/L phytosterols [18]. However, when the phytosterol concentration rose to 10 g/L, the concentration of 9-OHBA sharply decreased to 2.73 g/L. Therefore, a strain with a higher yield and selectivity of 9-OHBA needs to be developed.

**Figure 1:**
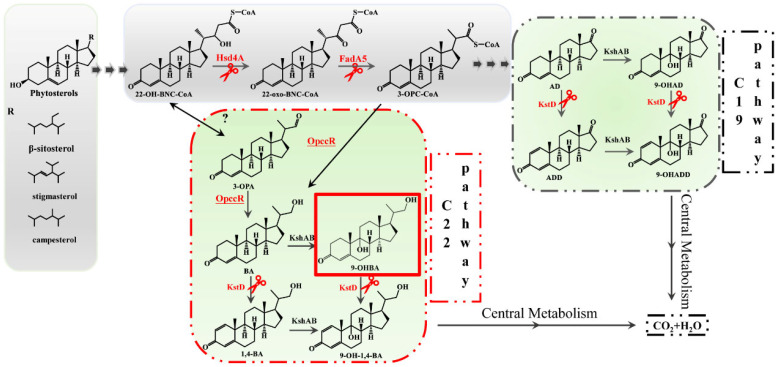
The predicted metabolic pathway of phytosterols in *Mycobacterium fortuitum*. The C19 pathway and C22 pathway are annotated and framed by black and red dotted lines, respectively. All edited genes are emphasized in red color. The question mark means that the enzyme catalyzing this reaction is not yet known in *Mycobacterium fortuitum*. Phytosterols consist of β-sitosterol, campesterol, and stigmasterol, which differ in their R groups. The diagram was drawn based on our previous article [16]. 22-OH-BNC-CoA, 22-hydroxy-3,24-dioxo-4-ene-cholest-CoA; 22-oxo-BNC-CoA, 3,22,24-trioxo-4-ene-cholest-CoA; 3-OPC-CoA, 3,22-dioxo-4-ene-pregna-CoA; 3-OPA, 3-oxo-4-ene-pregna-20-carboxyaldehyde; BA, 22-hydroxy-23, 24-bisnorchol-4-en-3-one; 1,4-BA, 22-hydroxy-23,24-bisnorchol-1,4-dien-3-one; 9-OHBA, 9,22-dihydroxy-23,24-bisnorchol-4-ene-3-one; AD, 4-androstene-3,17-dione; ADD, 1,4-androstadiene-3,17-dione; 9-OHAD, 9α-hydroxy-4-androstenedione; KshAB, 3-ketosteroid-9α-hydroxylase; KstD, 3-ketosteroid-Δ^1^-dehydrogenase; OpccR, a dual-function reductase; Hsd4A, 17β-hydroxysteroid dehydrogenase/β-hydroxyacyl CoA dehydrogenase; FadA5, acetyl-CoA-acetyltransferase/thiolase.

In our previous work, we solved the problem of the degradation of 9-OHBA and 9-OHAD by knocking all putative *kstds* in *Mycobacterium fortuitum* ATCC 35855 named MFKD in this study [19]. The mutant strain MFKD accumulated mainly 9-OHAD and 9-OHBA as a by-product. Therefore, to construct a 9-OHBA-producing strain, we tried to identify the key enzymes, as shown in Figure 1, that control metabolic flow between the C22 and C19 pathways. We bioinformatically identified one *hsd4A* gene, one *fadA5* gene, and one *opccR* gene in *Mycobacterium fortuitum* ATCC 35855 that were mainly responsible for switching the C19 and C22 pathways. After multiple genetic modifications, the results showed that blocking the C19 pathway increased the production of 9-OHBA. Our results also contributed to the knowledge of the complexity and diversity associated with regulating steroid catabolism in *M. fortuitum*. They provided a theoretical basis for the optimization of industrial microbial biocatalysts.

## 2. Results

### 2.1. Construction of Phylogenetic Tree of Hsd4A, FadA5, and OpccR

**Figure 2:**
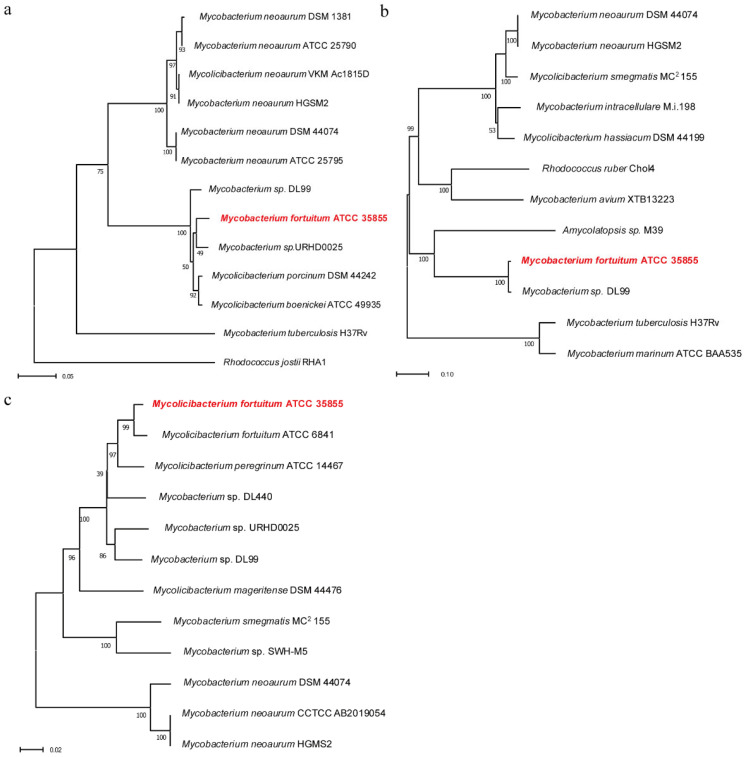
Phylogenetic trees of Hsd4A, FadA5, and OpccR in *Mycobacterium fortuitum* ATCC 35855 and other representative homologues. *Mycobacterium fortuitum* ATCC 35855 is highlighted in red color. (**a**) Phylogenetic tree of Hsd4A; (**b**) phylogenetic tree of FadA5; (**c**) phylogenetic tree of OpccR.

Whole-genome sequencing identified one putative *hsd4A*(gene1030), one putative *fadA5*(gene5404), and one putative *opccR* (gene1719) in *M. fortuitum* ATCC 35855. We translated the nucleotide sequences of the genes into amino acid sequences, and then, aligned them with their homologues, whose physicochemical properties and role in steroid metabolism were examined. The amino acid sequence of Hsd4A in *M. fortuitum* ATCC 35855 shared an identity of 78.8% with Hsd4A in *M. neoaurum* DSM 44074 [18], 78.5% with Hsd4A in *M. neoaurum* HGMS2 [20], 77.5% with Hsd4A in *M. neoaurum* DSM 1381 [21], and 69.3% with Hsd4A in *M. tuberculosis* H37V [13], whose Hsd4A enzymes were reported to have catalytic activities. As shown in Figure 2, their amino acid sequences shared a high similarity, while Hsd4A in *M. fortuitum* ATCC 35855 did not, which suggested a new unidentified Hsd4A.

The amino acid sequence of FadA5 of *M. fortuitum* ATCC 35855 shared a high identity of 88.9%, 88.4%, and 81.6% with FadA5 of *M. neoaurum* HGMS2 [20], *M. neoaurum* DSM 44074 [18], and *M. tuberculosis* H37V [12,22], respectively. Moreover, the alignments of the amino acid sequence of FadA5 confirmed that FadA5 has a conserved set of catalytic residues. The key amino acids at the active site, including Cys93, acted as nucleophiles attacking the steroid acyl-CoA’s β-keto carbonyl moiety. Additionally, Cys377 and His347 serve as regular acid/base residues. These results were consistent with Lu’s research in 2017 [22].

According to previous reports, OpccR was found in both *M. neoaurum* HGMS2 and *M. neoaurum* CCTCC AB2019054 and was reported to have catalytic activity in the steroid metabolic pathway [5,20]. One putative OpccR in *M. fortuitum* ATCC 35855 was found by sequence alignment, and shared 76.3% identity with those in *M. neoaurum* HGMS2 and *M. neoaurum* CCTCC AB2019054. A dendrogram of OpccR was also built and was chosen to analyze and speculate the function of Opccr in steroid metabolism. The alignment results revealed that the NADPH-binding motif in the C terminal domain (S375AA-G377AA-R398AA) was conserved.

### 2.2. Hsd4A—The Key Enzyme in the C19 Pathway 

**Figure 3:**
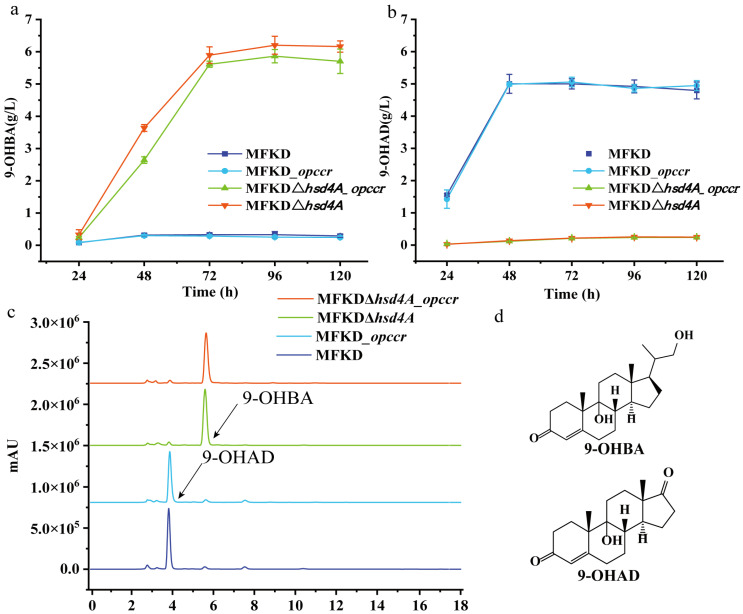
Phenotypic analyses of the metabolites of phytosterol produced by MFKD and its mutants. (**a**,**b**) Time course of 9-OHBA and 9-OHAD accumulation in mutant strains MFKD, MFKD*_opccR*, MFKDΔ*hsd4A*, and MFKDΔ*hsd4A_opccR*; (**c**) high-performance liquid chromatography (HPLC) elution profiles of metabolites produced via phytosterol bioconversion by MFKD, MFKD*_opccR*, MFKDΔ*hsd4A*, and MFKDΔ*hsd4A_opccR*; (**d**) structure of 9-OHBA and 9-OHAD. Error bars represent standard deviations.

As is shown in Figure 1, *hsd4A* controls the direction of metabolic flux to the C19 pathway by catalyzing 22-OH-BNC-CoA to form 22-oxo-BNC-CoA. So, herein, we constructed a strain named MFKDΔ*hsd4A* by knocking out *hsd4A* in MFKD. As predicted, the HPLC elution profiles showed that metabolic flow was significantly twisted, as shown in Figure 3c. A total of 81.47% 9-OHBA was accumulated compared with 4.13% in the strain MFKD, which confirmed the predicted function of *hsd4A* (Table 1). Moreover, the selectivity of 9-OHAD reduced to 4.07% from 78.09%. A concentration of 6.16 g 9-OHBA per liter was reached from 10 g/L phytosterols, and the molar yield was 72.74%.

As shown in Figure 1, reductase OpccR has activities in both 3-OPC-CoA and 3-OPA according to Peng’s study [5]. It turned out that Peng’s and Song’s studies came to opposite conclusions regarding the role of *mnopccR* in *Mycobacterium neoaurum* [5,20]. Enlighted by their research, in *M. fortuitum* ATCC 35855, we also found an *mnopccr* (designated *opccR* in this study) that shared 76.3% identity with *mnopccr* in *M. neoaurum* CCTCC AB2019054 using amino acid BLAST. To test the function of OpccR in *M. fortuitum* ATCC 35855, the strains MFKD*_opccR* and MFKDΔ*hsd4A_opccR* were constructed by overexpressing *opccR*. However, after the inducement of phytosterols, there were no significant differences between the mutant strains and original strains, as shown in Figure 3. The selectivity of 9-OHBA slightly increased to 4.76% in MFKD*_opccR* from 4.21% in MFKD by 13.06%, and 9-OHAD reduced to 77.98% from 78.09% (Table 1). 

These data indicated that the activity of the OpccR enzyme was much weaker than that in *Mycobacterium neoaurum*. In conclusion, the mutant strain MFKDΔ*hsd4A* was a good 9-OHBA-producing strain, although the by-product 9-OHAD was still present.

### 2.3. Deletion of fadA5 Improves the Selectivity of 9-OHBA by Eliminating 9-OHAD

**Figure 4:**
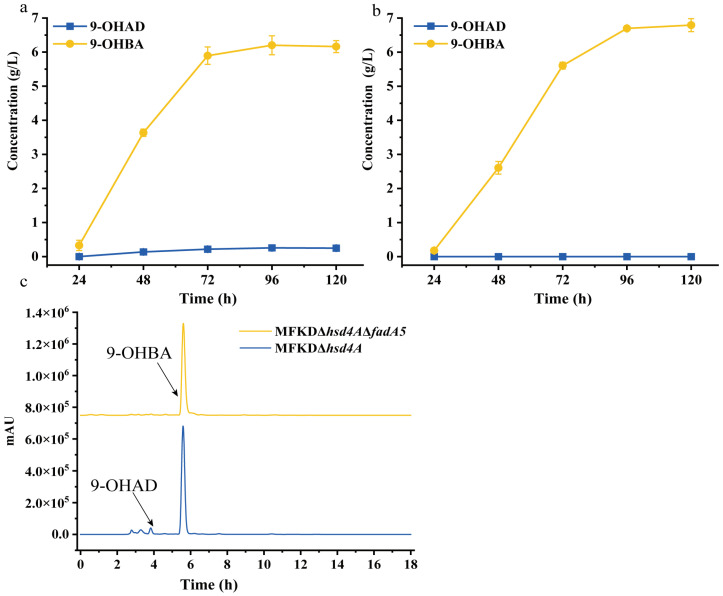
Phenotypic analyses of the metabolites of phytosterol produced by the 9-OHBA-producing strain. (**a**,**b**) Time profiles of product accumulation of mutant strains MFKDΔ*hsd4A* and MFKDΔ*hsd4A*Δ*fadA5*, respectively; (**c**) high-performance liquid chromatography (HPLC) elution profiles of metabolites produced via phytosterol bioconversion by MFKDΔ*hsd4A* and MFKDΔ*hsd4A*Δ*fadA5*. Error bars represent standard deviations.

The accumulation of 9-OHAD in the MFKDΔ*hsd4A* strain suggested that the C19 pathway of phytosterol degradation was not completely blocked. In order to further enhance the proportion of 9-OHBA and remove impurities, we deleted *fadA5* in MFKDΔ*hsd4A* to construct MFKDΔ*hsd4A*Δ*fadA5.* In line with our expectation, the double mutant MFKDΔ*hsd4A*Δ*fadA5* reduced 9-OHAD from 4.19% to 0.90%. The proportion of 9-OHBA reached 95.13%, which is an improvement of 13.62% compared with the former strain. The molar yield of 9-OHBA reached 80.33%. A concentration of 6.81 g 9-OHBA per liter was reached in MFKDΔ*hsd4A*Δ*fadA5* after it was cultured with 10 g/L phytosterols, as shown in Figure 4.

### 2.4. Evaluation of the 9-OHBA-Producing Strain

As 9-OHAD production was almost eliminated in the double mutant MFKDΔ*hsd4A*Δ*fadA5*, we performed high-concentration fermentation using this strain. To assess the potential of the 9-OHBA-producing strain for transforming phytosterols into 9-OHBA, the strain MFKDΔ*hsd4A*Δ*fadA5* was cultured in the fermentation medium with 10, 15, and 20 g/L phytosterols.

**Figure 5:**
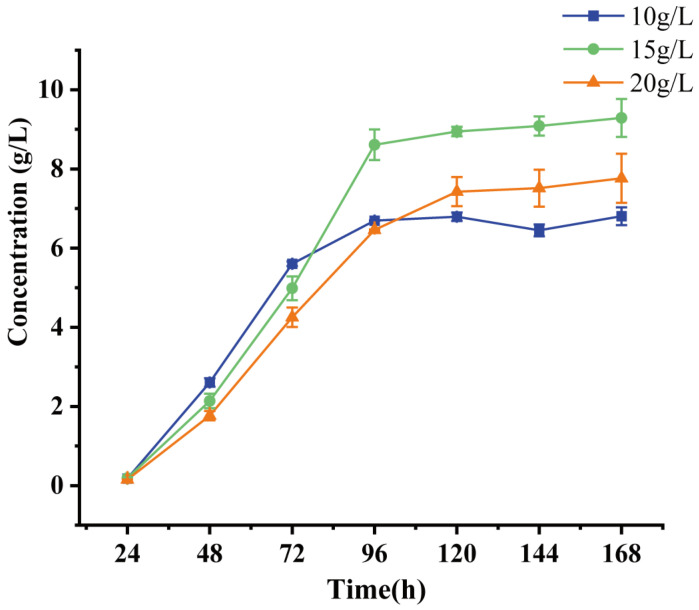
Time profiles of 9-OHBA accumulation by MFKDΔ*hsd4A*Δ*fadA5* with different concentrations of phytosterols. Error bars represent standard deviations.

As shown in Figure 5, we obtained 6.81 g/L, 9.28 g/L, and 7.76 g/L 9-OHBA from 10, 15, and 20 g/L phytosterols, respectively. The molar yields of 9-OHBA were 80.33%, 73.07%, and 45.82%, respectively. The selectivity of the main 9-OHBA product decreased to 90.98% when the concentration of phytosterols reached 15 g/L and was 86.92% when cultured with 20 g/L phytosterols. Meanwhile, the by-product of 9-OHAD increased to 3.80% when the concentration of phytosterols reached 20 g/L. 

## 3. Discussion

*Mycobacterium fortuitum* ATCC 35855 is a fast-growing strain and an outstanding producer of 9-OHAD. The mutant strain MFKD knocked out of five putative *kstDs* in our previous work prevented the degradation of the main nucleus to a large extent and finally accumulated 9-OHAD primarily and 9-OHBA as a by-product [19]. Owing to the extensive and in-depth research ons genes related to the C19 and C22 pathway in mycobacterial strains and the irreplaceable role of 9-OHBA in the synthesis of corticosteroids, we chose MFKD as a starting strain to obtain 9-OHBA and to find out the functions of related genes in *Mycobacterium fortuitum*.

Hsd4A, characterized as an enzyme that catalyzes 22-OH-BNC-CoA to form 22-oxo-BNC-CoA, is always chosen to regulate metabolic flux to produce C19 and C22 steroid intermediates [17]. In MFKD, we found one putative gene, *hsd4A*, whose product’s amino acids shared an identity of 78.8% with Hsd4A in *M. neoaurum* DSM 44074, which has been reported to have catalytic activities, indicating that it is a putative dehydrogenase [18]. Therefore, we knocked out *hsd4A* in the mutant strain MFKD, and the direction of metabolic flux twisted to the C22 pathway as expected. The mutant strain MFKDΔ*hsd4A* accumulated 9-OHBA as the main product with a selectivity of 81.47%. However, 4.07% of the by-product of 9-OHAD still remained, indicating that isozymes of Hsd4A still exist. MnOpccr was first identified in *M. neoaurum* CCTCC AB2019054 as a bifunctional enzyme that catalyzes both the 4-e reduction of 3-OPC-CoA by the C terminal domain and the 2-e reduction of 3-OPA to form 4-BA by the N terminal domain [5]. The research also found that the inactivation of *mnOpccr* can eliminate BA, while the overexpression of *mnOpccr* with *hsd4A* inactivation can result in the sole production of BA from phytosterols [5]. Therefore, in order to improve metabolic flux in the C22 steroid pathway and eliminate impurities, we decided to enhance the expression of OpccR. 

Given that OpccR has catalytic activities in both 3-OPC-CoA and 3-OPA, we constructed the mutant strains MFKD*_opccR* and MFKDΔ*hsd4A_opccR.* However, in this study, its overexpression neither in the MFKD strain nor in the mutant strain MFKDΔ*hsd4A* resulted in an evident increase in 9-OHBA. Specifically, the selectivity of 9-OHBA slightly increased by 13.06% to 4.76% in MFKD*_opccR* from 4.21% in MFKD. Given that the N and C terminal domains of OpccR are in charge of different reactions, OpccR in this MFKD may have a substrate preference for 3-OPC-CoA over 3-OPA. Another example is in *M. neoaurum* HGMS2. The OpccR enzyme was found to inhibit the accumulation of BA in HGMS2 [20]. This result contradicted Peng’s report, suggesting a different metabolic pathway. The limitation in catalytic activity and relatively low protein expression level of OpccR also contribute to this phenomenon. In addition, other isoenzymes may be present in this strain, the functions of which require further investigation.

FadA5 has been reported to cleave 3,22-dioxo-chol-4-ene-24-oyl-CoA to yield 3-OPC-CoA and Ac-CoA, and prefers the steroid CoA substrate [12]. In MFKD, we identified a putative gene, *fadA5*, which coded the thiolase FadA5. The amino acids of FadA5 shared an identity of 88.4% with *M. neoaurum* DSM 44074. As shown in Figure 1, we speculated that the knock-out of *fadA5* would further remove the by-product 9-OHAD. Hence, we inactivated FadA5 from MFKDΔ*hsd4A* to construct MFKDΔ*hsd4A*Δ*fadA5.* As expected*,* the selectivity of 9-OHAD reduced from 4.07% to 0.90%. Meanwhile, the target product 9-OHBA reached 95.13% from 81.47%, suggesting that the C19 pathway was blocked to a large extent. Finally, we obtained 6.81 g/L 9-OHBA after culturing with 10 g/L phytosterols for 144h using the mutant strain MFKDΔ*hsd4A*Δ*fadA5*. This yield ranks highest among the reported strains, compared with 3.58 g/L 9-OHBA from 5 g/L phytosterols and 2.73 g/L 9-OHBA from 10 g/L phytosterols from Yuan’s article [18]. The remaining 9-OHAD suggests that isozymes of Hsd4A and FadA5 still exist.

Finally, we obtained 6.81 g/L 9-OHBA with a selectivity of 95.13% and a molar yield of 80.33% after culturing with 10 g/L phytosterols for 144 h using the mutant strain MFKDΔ*hsd4A*Δ*fadA5*. This yield and selectivity rank highest among the strains reported so far using the shake flask fermentation method. Considering the best performance of the mutant strain MFKDΔ*hsd4A*Δ*fadA5* in producing 9-OHBA, we further evaluated its capacity under high concentrations of phytosterols. The concentration of 9-OHBA reached 9.28 g/L, which was highest when the strain MFKDΔ*hsd4A*Δ*fadA5* was cultured with 15 g/L phytosterols. However, the molar yield of 9-OHBA reduced to 73.07%. Only 7.76 g/L 9-OHBA was obtained when cultured with 20 g/L phytosterols, with a poor molar yield of 45.82%. Several reasons could be responsible for this phenomenon. Firstly, due to the low water solubility of phytosterols, their dispersion in aqueous phase is poor, thus limiting the accessibility of mycobacteria cells during phytosterol biotransformation [23]. The dispersibility of phytosterols further deteriorates with an increase in concentration. Therefore, dispersants such as surfactants, CDs (cyclic oligosaccharides), and water-miscible organic solvents are added to increase the solubility of the hydrophobic sterols in aqueous media. The ADD production of *M. neoaurum* VKPM Ac-1656 increased using the surfactant Tween-80 and the over-crosslinked polystyrene resin MN-200. Other surfactants like lecithin, polyoxyethylene (10) nonylphenyl ether (TX-40), and sucrose ester DK-Ester P-160 also proved to promote the transformation of phytosterols [24,25,26]. In addition, phytosterols and their derivatives inhibited cell growth by reducing the utilization of carbon sources and further hindered bioconversion [27]. Also, this effect became more prominent with an increase in phytosterol concentration. Poor fermentation conditions and low cell concentration in the shake flask also inhibited conversion efficiency. Large fermenters and high-oxygen conditions can alleviate this situation accordingly.

## 4. Materials and Methods

### 4.1. Bacterial Strains, Plasmids, Reagents, and Culture Conditions

The *Mycobacterium* strains and plasmids used in this study are listed in Table 2. The mutant *Mycobacterium* strains were constructed based on the strain MKFD [19], a KstD-deficiency strain derived from *Mycobacterium fortuitum* ATCC 35855 maintained in our laboratory. We employed the homologous recombinant knockout plasmid pKADel (obtained by combining p2NIL and pGoal19) and the P40 gene integrative vector, which was derived from the plasmid pMV306, for the gene modification of *M. fortuitum* [19].

LBT medium (10.0 g/L NaCl, 10.0 g/L tryptone, 5.0 g/L yeast extract, and 2.0 g/L Tween-80 (pH 7.0)) was used for aerobic cultivation of *M. fortuitum* at 30 °C and 200 rpm. MT medium, which contained 20 g/L glucose, 12 g/L (NH4)_2_HPO_4_, 0.5 g/L MgSO_4_, 0.5 g/L NaNO_3_, 3 g/L citric acid, 0.05 g/L ammonium ferric citrate, and 0.2% Tween-80 (*v*/*v*), was used as a fermentation medium by *Mycobacterium* cells. A total of 3mL seed medium was transferred to 30 mL of MT medium in a 250 mL shaker flask with a baffle when the optical density reached the mid-log exponential phase. Shaker flasks with baffles were used to increase the dissolved oxygen level in the culture medium effectively and enhance the contact between cells and air. The initial pH was adjusted to 7.5 and the fermentation was carried out at 30 °C and 220 rpm. The phytosterols consisted of 45% β-sitosterol, 37% campesterol, and 18% stigmasterol, purchased from Yunnan Biological Products Co., Ltd. (Kunming, China). A total of 60 g/L phytosterol mother liquor was configured by mixing phytosterol and (2-hydroxypropyl)-β-cyclodextrin (HP-β-CD) in water with a ratio of 1:3 (m/m), stirring for 15 min, ultrasound dispersing for 20 min, and repeating three times. AD, ADD, BA, 1,4-BA, 9-OHBA, and 9-OHAD were purchased from Sigma-Aldrich (Shanghai, China); all other reagents used were of analytical grade or higher unless noted otherwise.

### 4.2. Bioinformatic Analysis

The genome of ATCC 35855 has been sequenced previously and deposited in the GenBank database under the accession number CP110127 [19]. The putative genes for *hsd4A*, *fadA5*, and *opccR* were identified by comparison with known gene sequences from the NCBI database. Amino acid sequences of putative Hsd4A, FadA5, and OpccR were aligned by ClustalW; phylogenetic trees were constructed by neighbor-joining algorithm using MEGA 11 software.

### 4.3. Construction of Mutant Strains

Gene deletion and overexpression strategies were used to construct recombinant MFKD mutants. PKADel, obtained by combining p2NIL and pGoal19 [19,28], was used as the knockout plasmid via homologous recombination (Figure S1). Specifically, we amplified DNA sequences 1.2kb in length, located upstream and downstream of the target gene from the MFKD genome, including *hsd4A* and *fadA5* (Table S1). Then, the fragments were joined into linearized pKADel digested with AflII and SalI, constructing pKADelΔ*hsd4A* and pKADelΔ*fadA5.* Afterward, we electroporated the knockout plasmid into competent mycobacterial cells following previous procedures [19]. Finally, we screened the positive colonies using primers located upstream and downstream. The colonies with shorter fragments were confirmed to be positive. The vector p40 (pMV306 with the Psmyc promoter) was used to overexpress related genes in the mutant strains. *Opccr* was amplified from *M. fortuitum* ATCC 35855, and then, inserted into linearized P40 digested with AflII and HindIII to construct p40-*opccR*. Next, the plasmid was electroporated into the same competent mycobacterial cells as before. 

**Table 2:** Strains and plasmids used in this study.

Name	Description	Source
Strains		
*Escherichia coli*	*E. coli* DH5α	Vazyme Biotech Co., Ltd., Nanjing, China
MFKD	9-OHAD producer, *kstD1*&*2*&*3*&*4*&*5* deletion mutant of ATCC 35855	Our lab
MFKD*_opccR*	ATCC 35855 *opccR* overexpression in MFKD via p40-*opccR*	This study
MFKDΔ*hsd4A*	*hsd4A* deletion mutant of MFKD	This study
MFKDΔ*hsd4A_ opccR*	ATCC 35855 *opccR* overexpression in MFKDΔ*hsd4A* via p40-*opccR*	This study
MFKDΔ*hsd4A*Δ*fadA5*	*hsd4A* and *fadA5* double-deletion mutant of MFKD	This study
Plasmids		
pKADel	Plasmid for allelic exchange, Pag85-lacZ Phsp60-sacB, AprR, KanR	[19]
pKADelΔ*hsd4A*	pKADel carrying two homologous arms of *hsd4A*	This study
pKADelΔ*fadA5*	pKADel carrying two homologous arms of *fadA5*	This study
p40	pMV306 with Psmyc promoter, KanR	[19]
p40-*opccR*	p40 possessing *opccR* from *M. fortuitum* ATCC 35855	This study

### 4.4. Bioconversion and Analytical Methods

The mutant strains were first cultured in the LBT medium to the mid-logarithmic growth stage, and then, transferred to the MT medium containing phytosterol at 10% (*v*/*v*). The initial concentration of phytosterol was 10 g/L. Later, a concentration gradient was tested to further determine the strains’ ability for phytosterol bioconversion. An appropriate amount of evenly dispersed phytosterol mother liquor was taken and diluted with MT medium to the desired concentration to obtain the different fermentation concentrations of phytosterol.

In the *M. fortuitum* mutant fermentation experiments, samples were taken every 24 h for 5–7 days, and three replicates were used to measure and quantify steroids. Culture samples (0.5 mL) were extracted on a vortex mixer with 1 mL of ethyl acetate for 20 min before centrifugation at 12,000× *g* for 1 min. For HPLC, the organic phase of the sample was redissolved in methanol after volatilizing, and then, filtered through a 0.22 μm microporous membrane. Separation was performed on an Agilent XDB-C18 column (4.6 × 250 mm; 40 °C), and a UV/visible detector (254 nm) was employed to detect the steroid substrate conversion rates with methanol/water (80:20, *v*/*v*). The flow rate was 0.8 mL/min.

The molar yield (*My*) of steroid products 9-OHAD and 9-OHBA was calculated using the following equation:

$$My=\frac{Ma}{Mt}\times 100\%,$$

where *Ma* and *Mt* are the moles of actual steroid products and theoretical steroid products, respectively.

## 5. Conclusions

In this study, after knocking out *hsd4A* and *fadA5*, we successfully constructed an ideal 9-OHBA producer, MFKDΔ*hsd4A*Δ*fadA5*, with high yield and selectivity. This result proves that Hsd4A and FadA5 are crucial enzymes controlling the C19 metabolic pathway of steroids in *Mycobacterium fortuitum* ATCC 35855. But the function of OpccR in *M. fortuitum* ATCC 35855 still requires further research. Our work provides new insights into the strategies and methods for the production of relevant steroid intermediates.

## Figures and Tables

**Table 1:** The relative selectivity of the steroid intermediates produced by MFKD and its mutants.

Strain	Relative Selectivity (%)			
	9-OHAD	9-OHBA	AD	BA
MFKD	78.09 ± 0.21	4.21 ± 0.08	0.82 ± 0.01	0.09 ± 0.01
MFKD*_opccR*	77.98 ± 2.06	4.76 ± 0.31	0.87 ± 0.16	0.12 ± 0.03
MFKDΔ*hsd4A*	4.07 ± 0.12	81.47 ± 0.04	0.35 ± 0.01	0.21 ± 0.01
MFKDΔ*hsd4A_ opccR*	4,67 ± 0.13	81.22 ± 0.37	0.63 ± 0.22	0.19 ± 0.01
MFKDΔ*hsd4A*Δ*fadA5*	0.90 ± 0.08	95.13 ± 0.46	0.37 ± 0.08	0.19 ± 0.02

## Data Availability

The genome sequencing information of *M. fortuitum* ATCC 35855 has been deposited in the GenBank database with accession number CP110127. The accession numbers of the *hsd4A*, *fadA5*, and *opccR* gene sequences are OP729274.1, WP_019512519.1, and OP729275.1, respectively.

## Supplementary Materials

The following supporting information can be downloaded at: https://www.mdpi.com/article/10.3390/ijms25073579/s1.
