# Supplementary material for: Efficient Production of 9,22-Dihydroxy-23,24-bisnorchol-4-ene-3-one from Phytosterols by Modifying Multiple Genes in Mycobacterium fortuitum
Source: Int J Mol Sci. 2024 Mar 22;25(7):3579. doi: 10.3390/ijms25073579 (PMC11011972; doi:10.3390/ijms25073579)
Supplement: Supplementary file 1 [file ijms-25-03579-s001.zip › ijms-2917301-supplementary.pdf]

Table S1 Primers used in this study.

Name	Nucleotide
Primers for gene deletion	
hsd4A-U-F	atagaatacataggatccgtcgactggtgacgagcttccagcccgcg
hsd4A-U-R	tgcagtgccccgctgaggtcgatggcttcggcacggccgagg
hsd4A-D-F	tcggccgtgccgaagccatcgacctcagcggggcactgcacgat
hsd4A-D-R	cgttgttgccattgctgcagcttaagcggcggaagcggagtgtggcg
fadA5-U-F	tatagaatacataggatccgtcgacccacacgctctccggccttgatcttgac
fadA5-U-R	ttgtcggtgcgctccagctcgaggagctcggtggcgtggaggcc
fadA5-D-F	tccacgccaccgagctcctcgagctggagcgcaccgacaagagcacg
fadA5-D-R	cgttgttgccattgctgcagcttaagtggccgccgcgaacagtgaacatgc
Primers for gene overexpression and verification	
hsd4A-F	atgaccactgacgacgctca
hsd4A-R	tcagtctcgtgacgccatca
fadA5-F	atgggtaaccctgtcatcgtcg
fadA5-R	tcagatccgctcgatgatggt
p40-opccR-F	ccatcaggaggaatcctgcatgaccgacatgcattatgtcgttac
p40-opccR-R	gtgcgaagtgattcctccgcttaagttaccagtgcacacccggaatcg
p40-F	attcgccgcccgaaatgag
p40-R	ttgatgcctggcagtcgatcg

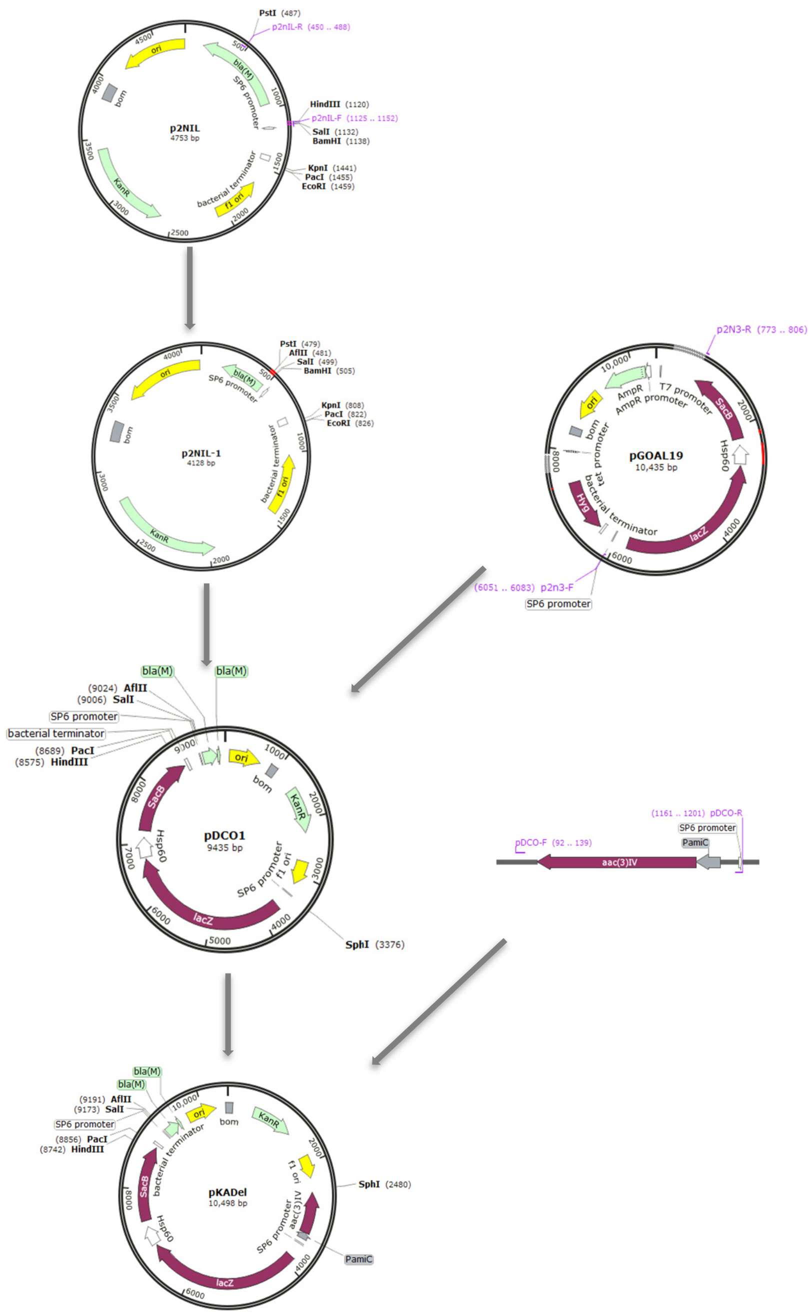

Figure S1 Construction of suicide plasmid pKADel.
